# Retraction for Lkb1﻿﻿ deletion in periosteal mesenchymal progenitors induces osteogenic tumors through mTORC1 activation

**DOI:** 10.1172/JCI204387

**Published:** 2026-02-02

**Authors:** Yujiao Han, Heng Feng, Jun Sun, Xiaoting Liang, Zhuo Wang, Wenhui Xing, Qinggang Dai, Yang Yang, Anjia Han, Zhanying Wei, Qing Bi, Hongbin Ji, Tiebang Kang, Weiguo Zou

Original citation: *J Clin Invest*. 2019;129(5):1895–1909. https://doi.org/10.1172/JCI124590

Citation for this retraction: *J Clin Invest*. 2026;136(3):e204387. https://doi.org/10.1172/JCI204387

The Editors recently became aware of errors in Figure 3E, Figure 7E, Supplemental Figure 2C, and Supplemental Figure 6B. Although the authors have indicated that original source data support the findings in these figure panels, the Editors are retracting the article due to the number of errors made.

Yujiao Han, Heng Feng, Jun Sun, Xiaoting Liang, Zhuo Wang, Wenhui Xing, Qinggang Dai, Yang Yang, Anjia Han, Zhanying Wei, Qing Bi, Hongbin Ji, Tiebang Kang, and Weiguo Zou dissent from the Journal’s decision to retract.

## Version 1 02/02/2026

Electronic publication

